# Neuropsychiatric Lyme Borreliosis: An Overview with a Focus on a Specialty Psychiatrist’s Clinical Practice

**DOI:** 10.3390/healthcare6030104

**Published:** 2018-08-25

**Authors:** Robert C. Bransfield

**Affiliations:** Department of Psychiatry, Rutgers-Robert Wood Johnson Medical School, Piscataway, NJ 08854, USA; bransfield@comcast.net; Tel.: +1-732-741-3263; Fax.: +1-732-741-5308

**Keywords:** Lyme disease, *Borrelia burgdorferi*, tickborne diseases, persistent infection, treatment, assessment, depression, anxiety, sleep disorders, opioid addiction

## Abstract

There is increasing evidence and recognition that Lyme borreliosis (LB) causes mental symptoms. This article draws from databases, search engines and clinical experience to review current information on LB. LB causes immune and metabolic effects that result in a gradually developing spectrum of neuropsychiatric symptoms, usually presenting with significant comorbidity which may include developmental disorders, autism spectrum disorders, schizoaffective disorders, bipolar disorder, depression, anxiety disorders (panic disorder, social anxiety disorder, generalized anxiety disorder, posttraumatic stress disorder, intrusive symptoms), eating disorders, decreased libido, sleep disorders, addiction, opioid addiction, cognitive impairments, dementia, seizure disorders, suicide, violence, anhedonia, depersonalization, dissociative episodes, derealization and other impairments. Screening assessment followed by a thorough history, comprehensive psychiatric clinical exam, review of systems, mental status exam, neurological exam and physical exam relevant to the patient’s complaints and findings with clinical judgment, pattern recognition and knowledgeable interpretation of laboratory findings facilitates diagnosis. Psychotropics and antibiotics may help improve functioning and prevent further disease progression. Awareness of the association between LB and neuropsychiatric impairments and studies of their prevalence in neuropsychiatric conditions can improve understanding of the causes of mental illness and violence and result in more effective prevention, diagnosis and treatment.

## 1. Background 

Lyme borreliosis (LB) is caused by *Borrelia burgdorferi* and other *Borrelia* species, such as *Borrelia garinii* and *Borrelia afzelii*. Other tick-borne and opportunistic infections may be present as well [1]. The most common co-infections seen with LB include *Anaplasma phagocytophilum, Babesia* species, *Ehrlichia chaffeensis*, *Rickettsia* species and *Bartonella* species; but there are also other less common and yet unknown pathogens [1]. Secondary co-infections or opportunistic infections are also commonly seen in patients with LB [1]. There is increasing evidence and recognition that LB causes mental symptoms, although debate exists regarding the role of LB vs. other tick-borne diseases (TBD) in the pathogenesis of neuropsychiatric symptoms [2]. Currently there are over 400 peer-reviewed articles addressing different aspects of neuropsychiatric symptoms caused by LB [2].

Although mental illnesses have been categorized based upon symptoms and syndromes since 1952 by the American Psychiatric Association in Diagnostic and Statistical Manuals (DSM), this categorization does not address the actual cause of mental illnesses [3]. Mental illness is associated with an impairment of adaptive capabilities. The causes of these impairments need to be viewed from the perspective of the interaction of multisystemic contributors and deterrents and their impact upon disease progression over time. Less complex and better understood disease models consist of well-defined and more limited causes, pathophysiology and clinical presentations. The diseases that are more challenging to understand consist of multiple contributors, multiple pathophysiological pathways and multiple disease presentations.

Several developments have helped our understanding of complex disease, including the better recognition of the role of chronic infections in chronic disease and attention to the human microbiome and a significant cause of disease is trauma from competing organisms, such as microbes [4]. The Centers for Disease and Prevention of the United States (CDC) has stated that clinicians and policymakers must recognize that many chronic diseases may indeed have infectious origins [5]. The National Institute of Health Human Microbiome Project recognizes bacterial cells outnumber human cells by 10 to 1, humans depend on their microbiome and a person should really be considered a superorganism [6]. Although the Infectome is beneficial to health in many ways, there are thousands of articles demonstrating a causal association between infections and mental illness, especially viral, venereal and vector-borne diseases [2,7,8].

When mental hospitals were filled with syphilitic patients everyone eventually recognized infections caused mental illness. After penicillin helped control this epidemic there was a reduced attention to the association between infectious disease and mental illness. Subsequently, attention to concepts relating to the microbiome and psychoimmunology facilitated by microarray testing and further research reactivated attention to the role of infectious contributors to the pathogenesis of mental illness. There are currently over 100 different infectious agents and the immune reactions to them known to cause mental illnesses, including spirochetes, other bacteria, viruses, parasites, protozoa, yeasts, fungi and prions [7,8].

A number of infections, including LB has particularly significant models explaining the association between infections and the development of mental illness. Although there have been some prior neuropsychiatric LB general review articles, no other neuropsychiatric LB journal articles have been published recently and a more current review was needed. [7,9,10,11].

## 2. Materials and Method

Data for this article were drawn from a database maintained by the author that includes all the journal articles addressing LB and their association with neuropsychiatric symptoms, other articles and presentations on the subject and experience from treating thousands of LB patients over decades. These data were reviewed to look for current information on LB and neuropsychiatric symptoms. The medical literature was also reviewed with PubMed and Google Scholar searches for additional information in a number of categories which included pathophysiology, clinical presentations, assessment and treatment. Particular attention was given to valid research articles that calculated the prevalence of acquired neuropsychiatric findings in LB patients post infection. Since all these studies were performed with a different study design, each study design is described. Some statistics were drawn from the Aggressiveness, Violence, Homicidality, Homicide and Lyme Disease article since it contained data on psychiatric findings after acquiring LB [12]. These patients were diagnosed with a comprehensive assessment including premorbid assessment, history of tick exposure, history of the presence of erythema migrans rash, early tick-borne disease symptoms, symptoms in the course of illness, current status with a comprehensive neuropsychiatric and general medical assessment, review of systems using the 280-item Neuropsychiatric Lyme Assessment with pattern recognition, and a review of laboratory assessment, which may include enzyme-linked immunosorbent assay, immunofluorescent assay, Western blot, DNA-based testing, coinfection testing, single-photon emission tomography, magnetic resonance imaging, or other diagnostic testing to determine the diagnosis in compliance with the Institute of Medicine–recognized guidelines. In addition to diagnostic criteria, the patients who met CDC surveillance criteria were noted, which is more stringent criteria and captures less than 10% of LB cases [13,14,15,16]. When this data was used, the non-homicidal patient control group was designated as LB-control (LB-C) and the LB homicidal patient group was designated as LB-H. 74% of the LB-C patients met the CDC surveillance criteria and 66% of the LB-H patients met the CDC surveillance criteria (physician documented erythema migrans rash and/or positive serology). In this database the LB-homicidal patient group consisted of 50 LB patients who presented with different degrees of homicidality. Some in this group only had homicidal thoughts, impulses or behavior, while others committed homicides. There were 5 homicides committed by this group, all of whom met CDC surveillance criteria.

## 3. Results

### 3.1. Pathophysiology 

Many infections are associated with an early inflammatory reaction followed by adaptive immunity and a resolution of symptoms, but in some chronic infections that evade and suppress the immune system, such as *B. burgdorferi* and other *Borreliae*, inflammation can persist without adaptive immunity, autoimmune symptoms may occur, and relapses and reinfections are common. [2,17,18,19,20,21].

There are three basic types of *B. burgdorferi* infections causing neuropsychiatric symptoms—the meningovascular form associated with cerebrovascular infarcts; the second is infection within the central nervous system (CNS) which is the atrophic form of Lyme meningoencephalitis and is associated with cortical atrophy, gliosis and dementia and the third is infection outside the CNS causing immune and other effects within the CNS that contribute to neuropsychiatric symptoms. A LB patient with neuropsychiatric symptoms may have one or more than one of these three types of infections [22,23,24].

Although some injury to the host is a result of the direct action of the parasite upon the host, more often it is the immune reaction to the infection that results in symptoms in the host. Articles have described how the immune and psycho-immune responses to *B. burgdorferi* have resulted in psychiatric symptoms [2,24,25]. Immune mediated effects are significant contributors to the pathophysiological processes and disease progression. These immune effects include persistent inflammation with cytokine effects and autoimmunity and both of these mechanisms may occur at the same time in persistent infections [2,24,25].

Lyme disease has been associated with the proinflammatory cytokines Interleukin-6, Interleukin-8, Interleukin-12, Interleukin-18 and interferon-gamma; the chemokines CXCL12, CXCL13 and CCL19 and increased levels of proinflammatory lipoproteins [24,25,26,27,28]. *B. burgdorferi* surface glycolipids and flagellar proteins appear to elicit anti-neuronal antibodies and these *B. burgdorferi* surface glycolipids and flagella antibodies can disseminate from the periphery to inflame the brain resulting in persistent inflammatory effects that can be associated with neurodegenerative changes [24]. Persistent inflammation is also associated with metabolic changes provoked by these immune reactions and include oxidative stress, excitotoxicity, changes in homocysteine metabolism, mitochondrial dysfunction, altered tryptophan catabolism, decreased serotonin and increased quinolinic acid [24]. The presence of chronic inflammation is associated with increased proinflammatory cytokines which increase levels of indoleamine 2,3-dioxygenase, which converts tryptophan into quinolinic acid which is a neurotoxic metabolite and is a known agonist of *N*-methyl-D-aspartate synaptic function and increases depressive, cognitive and other symptoms [29]. Quinolinic acid is significantly elevated in cerebral spinal fluid in *B. burgdorferi* infections, more significantly in patients with CNS inflammation than in encephalopathy and correlates with the severity of CNS symptoms, including depression [27,30].

Besides *B. burgdorferi,* other known interactive tick-borne diseases such as *Babesia*, *Bartonella*, *Ehrlichia*, and *Mycoplasma* and unknown agents have immune and metabolic effects that further add to the complexity of the pathophysiology of tick-borne infections [31,32,33,34,35]. Opportunistic coinfections which may or may not be tickborne pathogens may also add to the complex interactive infectious process [36].

Some chronic symptoms are associated with injury and resulting dysfunction from past infection(s), other chronic symptoms are associated with chronic persistent or latent and relapsing infections [24]. In spite of considerable evidence supporting persistent infection with LB, some speculate that disease progression is caused by some continuing self-perpetuating post-infectious pathological process, although no viable mechanism has ever been demonstrated. However, what starts a disease process may be different from what causes further disease progression. Non-restorative sleep and chronic unremitting stress appear to play a significant role in disease progression in LB. In one study all LB patients meeting CDC surveillance criteria studied had acquired sleep-related complaints [37]. Both non-restorative sleep and the chronic unremitting stress seen in these chronically ill patients contribute to disease perpetuation and progression and are associated with fatigue, cognitive impairments, decreased regenerative functioning, compromised immunity, decreased resistance to infectious disease and neurodegenerative processes [38,39,40,41,42].

### 3.2. Clinical Presentations

It is recognized up to 40% of LB patients develop neurologic involvement of either the peripheral or central nervous system [10]. Similar to syphilis, LB may have a latency period of years before symptoms of late infection emerge. A broad range of psychiatric findings associated with LB include paranoia, dementia, schizophrenia, bipolar disorder, panic attacks, major depression, anorexia nervosa, and obsessive-compulsive disorder [10].

In reviewing multiple articles, it was apparent that each patient can have a unique and variable clinical presentation, however common symptom patterns are seen. Pre-infection most patients were young, quite active and healthy. A LB infection may have no or minimal effect in some, be severe in some, result in a latent infection in others, have a relapsing and remitting course in others, be slowly progressive in some and be rapidly progressive in others. It may cause a spectrum of multisystem symptoms which may include neuropsychiatric and somatic symptoms that may be initially subtle while becoming more severe with further disease progression. The neuropsychiatric manifestations may be cognitive, emotional, vegetative and behavioral and can be associated with almost any diagnosis in the DSM, but some psychiatric syndromes are more commonly seen than others. Significant psychiatric comorbidity is commonly seen. Infections at different times in the lifespan (congenital, infancy, childhood, adolescence, adulthood, geriatric) have different pathological effects [8,9,10,11].

Studies have looked at both the presence of LB in identified psychiatric patients and the emergence of neuropsychiatric symptoms in identified LB patients after becoming infected. In identified psychiatric patients, one study of 517 hospitalized psychiatric patients in a highly endemic area failed to find sufficient immune diagnostic reactivity to *B. Burgdorferi* meeting CDC surveillance criteria in a single patient [43], while another study of 1810 demonstrated a higher prevalence of antibodies to *B. burgdorferi* was seen in hospitalized psychiatric patients when compared to matched pairs of healthy subjects (33% vs. 19%) [44]. An outpatient retrospective chart review without a control group demonstrated 80% of children with psychiatric illness referred to a child psychiatrist, 49 of the 69 without a known history of LB or other TBD, demonstrated evidence of exposure to one or more of the pathogens *B. burgdorferi*, *Bartonella, Babesia*, *Ehrlichia* and *Anaplasma* on serologic testing performed by multiple laboratories and 22% of children with an onset of bipolar disorder referred to a child psychiatrist tested CDC surveillance criteria positive for LB. In addition, 74% of these children tested positive to exposure to one or more of the pathogens *B. burgdorferi*, *Bartonella, Babesia*, *Ehrlichia* and *Anaplasma* on serologic testing performed by multiple laboratories [45,46,47].

In identified LB patients a number of studies have looked at the percentages of different psychiatric findings that emerged post-infection. These studies were on patients who were mostly young and healthy pre-infection and there were studies in which the control group consisted of the same patients prior to infection [12,48]. The details of these studies shall be discussed further when discussing different disease presentations.

The total neuropsychiatric symptoms associated with LB and LB/TBD results in a significant amount of impairment, disability and death [12,48,49,50].

### 3.3. Developmental Disorders

Congenital LB infections can contribute to developmental disorders and neuropsychiatric impairments [51,52,53]. Congenital transmission of *Bartonella* has also been documented [48,49,50,51,52,53,54]. Since 1985 there are over 60 references documenting congenital transmission and associated pathological outcomes with LB/TBD [2,55]. The most comprehensive study was a review of 263 cases and included cases of miscarriage, stillbirth, perinatal death, congenital anomalies, systemic illness, early onset fulminant sepsis and later-onset chronic progressive symptoms associated with gestational LB [56].

The study most relevant to neuropsychiatric symptoms was a retrospective chart review of 102 gestational LB cases which were diagnosed by clinical criteria, Lyme enzyme-linked immunosorbent assay testing, Lyme Western blot testing, Lyme urine antigen testing, culture, polymerase chain reaction (urine), polymerase chain reaction (blood), single-photon emission computed tomography and magnetic resonance imaging. This study demonstrated 9% had been diagnosed with autism and 56% with attention deficit disorder in addition to a broad spectrum of multisystem symptoms. Other psychiatric symptoms included irritability or mood swings (54%), anger or rage (23%), anxiety (21%), depression (13%), emotional lability (13%), obsessive compulsive disorder (11%), suicidal thoughts (7%), developmental delays (18%), tic disorders (14%), seizure disorders (11%), involuntary athetoid movements (9%), photophobia (43%), auditory hyperacuity (36%), other sensory hypersensitivity (tactile, taste or smell) (23%), poor memory (39%), cognitive impairments (27%), speech delays (21%), reading/writing impairments (19%), articulation impairments (17%), auditory/visual processing impairments (13%), word selectivity impairments (12%), and dyslexia (18%). In the control group of 66 mothers with Lyme disease who were treated with antibiotics prior to conception and during the entire pregnancy: all gave birth to normal healthy infants. However, in the control group there were eight pregnancies that resulted in *B. burgdorferi* and/or *Bartonella henselae* positive placentas, umbilical cords, and/or foreskin remnants. The PCR positive cases were treated successfully with oral antibiotics [57].

### 3.4. Autism Spectrum Disorders

Autism spectrum disorder (ASD) results from multiple etiologies with both genetic and environmental contributions, including at least 23 different infections, seven of which are chronic infections (*Babesia*, *Bartonella*, *B. burgdorferi*, *Ehrlichia*, Human *Herpesvirus-6*, *Chlamydia pneumoniae* and *Mycoplasma*), and the immune reactions associated with these infections [7]. Skepticism of the association between LB and ASD is partially based upon using the CDC surveillance criteria as a sole diagnostic criterion [58] and the validity of this belief has been challenged with this group of patients [59]. ASD has been found to be associated with LB [7,57,59,60,61,62,63,64,65,66]. The timing of the infection and immune response is critical in determining the pathophysiology. In congenital infections maternal immune reactions to infections appear to adversely affect fetal brain development and possible pathophysiological mechanisms include both autoimmune and inflammatory processes [7,57]. The association between ASD and LB is often overlooked. One study demonstrated 94% of children with a diagnosis of ASD and LB initially tested negative on current two tier CDC *B. burgdorferi* surveillance criteria testing, however 92% of LB patients with ASD had reactivity of the highly specific 34 kilodalton (kDa) band (outer surface protein-B) and the less specific, but neurotropic and often significant 31 kDa band (outer surface protein-A) on Western blot testing which had previously been included in the CDC surveillance criteria and reported on the commercially available tests for *B. burgdorferi* [67]. After the Dearborn meeting in 1994 the 31 kDa and 34 kDa bands were removed from the CDC surveillance criteria, which facilitated the planned development of vaccines based upon these two antigens, but reduced the sensitivity of the CDC surveillance criteria and contributed to an underdiagnosis of some patients [67,68].

States in the United States with the highest prevalence of ASD have the highest prevalence of *B. burgdorferi* and states with the lowest prevalence of ASD have the lowest prevalence of *B. burgdorferi* [69].

Treatment of LB during pregnancy can prevent the development of ASD associated with LB [57]. Another study demonstrated antibiotic treatment can reduce symptoms of ASD associated with LB [69].

### 3.5. Schizophrenia and Schizoaffective Disorder

Schizophrenia has been associated with a number of infections rather than LB and evidence drawing an association between LB is limited [70,71]. There is, however, a significant geographical correlation between *Ixodes* ticks, LB and schizophrenia in the United States [72]. When schizophrenia is seen with LB, it is most commonly schizoaffective disorder [73,74,75,76,77,78,79]. In late stage LB patients, paranoid symptoms had an overall prevalence of 36% (LB-C) [12] and 88% (LB-H) [12] and hallucinations had an overall prevalence of 42% (LB-C) [12] and 47% (LB-H) [12].

### 3.6. Bipolar Disorder

Bipolar disorder has been associated with a number of infections including LB [46,73,80,81,82]. When bipolar disorder is seen in LB patients, it is invariably rapid cycling [12]. Mood lability has also been reported [83].

Different studies have demonstrated the prevalence of bipolar illness in LB at 22% (in children) by CDC surveillance criteria [47], 28% (LB-H) [12] and 10% (LB-C) [12].

### 3.7. Depression

Depression from LB can be reduced with early diagnosis and effective treatment [84,85]. However, when LB has not been adequately diagnosed and treated, it is a common finding. Studies of different groups with have shown a prevalence of depression of 0% pre-infection [12] and a post-infection incidence of 98% (LB-H) [12], 94% (children) (In this study the diagnosis of LB was confirmed based on (a) history of exposure to a Lyme endemic area, (b) an illness course distinguished by symptoms characteristic of LB, and (c) either (1) history of a physician-documented erythema migrans (EM) rash or unambiguous EM described by a parent, or (2) history of a positive whole-blood polymerase chain reaction test for *B. burgdorferi* or a positive Western blot meeting explicit current CDC surveillance criteria. The CDC surveillance criteria for the immunoglobulin G Western blot were broadened to recognize that the 31-kD and 34-kD bands represent the highly specific outer surface protein A and B bands.) [86], 80% (with intrusive symptoms) (From the same database in which 70% met CDC surveillance criteria [12]) [87], 76% (LB-C) [12] 64% (Based upon an online survey of respondents who were clinically diagnosed with LD and had persisting symptoms lasting more than 6 months following antibiotic treatment) [88], 51% (LB and neuroborreliosis was diagnosed in all patients on the basis of a standardized interview, physical examination and the results of laboratory tests, according to the current European criteria. Serological tests were performed using the enzyme-linked immunosorbent assay and confirmed using Western blotting. Neuroborreliosis was diagnosed on the basis of the cerebrospinal fluid test and proving intrathecal synthesis of anti-Borrelia burgdorferi antibodies.) [89], 50% (Among patients who had suffered from severe meningitis, meningoencephalitis or meningopolyradiculoneuritis due to neuroborreliosis in the chronic form of the illness) [90], 37% (A conference presentation in which all patients previously had signs of LB, had neurologic symptoms lasting at least three months that could not be attributed to another cause, and had current evidence of humoral or cellular immunity to *B. burgdorferi*, as shown by an elevated serum IgG or IgM antibody titer of at least 1:400, five or more IgG antibody bands to spirochetal polypeptides or a stimulation index of 10 or more in response to borrelial antigens.) [91] and 37% (children) (Patients met CDC surveillance criteria or had been diagnosed with an erythema migrans or both) [92].

### 3.8. Anxiety Disorders

Different types of anxiety may be caused by LB. An early manifestation of hyperarousal may present as hypervigilance (54%) (LB-C) [12] and (84%) (LB-H) and/or low frustration tolerance (80%) [48] and (98%) (LB-H) [12]. Further symptoms may then include mixed anxiety or different anxiety disorders, such as panic disorder, social anxiety disorder, generalized anxiety disorder, obsessive compulsive disorder and posttraumatic stress disorder.

Panic disorder has been associated with LB [12,75,80,92,93,94,95,96]. Panic disorder has demonstrated a prevalence of 82% (LB-H) [12], 54% (children) [92] and 50% (LB-C) [12].

Although no article has ever specifically addressed social anxiety disorder associated with LB, it is a common finding in patients. The prevalence of social anxiety disorder in LB has been demonstrated to be 70% (LB-C) [12] and 66% (LB-H) [12].

Generalized anxiety has been associated with LB and was 50% (From the same database in which 70% met CDC surveillance criteria [12]) [48] and 86% (LB-H) [12].

Obsessive compulsive disorder has been reported with LB, can have an autoimmune pathophysiology and can have a very sudden onset [95,96,97,98,99]. Based upon a survey of 147 patients who reported they had been diagnosed with LB the prevalence of obsessive compulsive symptoms in LB was 84% and 44% of these participants self-identified these symptoms as problematic and 51% reported at least some improvement in obsessive compulsive symptoms following antibiotic treatment. [100], 51% (LB-H) [12] and 32% (LB-C) [12].

Posttraumatic stress disorder has been associated with LB [87,101,102]. The prevalence of posttraumatic stress disorder was 24% (LB-C) [12] and 36% (LB-H) [12].

Intrusive Symptoms are associated with LB and may be present with obsessive compulsive disorder, posttraumatic disorder or be present without either of these conditions and demonstrated a prevalence of 34% and the intrusive symptoms included aggressiveness in 89%, altered sexual imagery in 18% and 40% had other intrusive symptoms including bizarre and horrific images [87]. In another study intrusive aggressive images were seen in 62% of (LB-H) patients [12] but in only 16% of (LB-C) patients [12]. Intrusive sexual images were seen in 26% of (LB-H) patients [12] while they were seen in only 6% of (LB-C) patients [12]. LB patients with intrusive symptoms also had cognitive impairments (100%), neurological symptoms (98%), obsessiveness (89%), depersonalization (87%), depression (80%), low frustration tolerance (80%), explosive anger (73%), suicidality (69%), social isolation (67%), anhedonia (62%), disinhibition (62%), paranoia (49%), hallucinations (42%) and homicidality (31%) [87].

### 3.9. Eating Disorders

A number of eating disorders are associated with LB. Some LB patients lose weight early in the disease process and later gain weight. Cases of anorexia nervosa, bulimia and excessive weight gain have been reported [10,103,104].

### 3.10. Sleep Disorders

Sleep disorders acquired as a result of LB are quite significant and include insomnia (early, mid, late), non-restorative sleep, restless leg, paroxysmal nocturnal leg movements, sleep apnea (obstructive and central), nightmares, circadian rhythm shift and narcolepsy (with sleep attacks, cataplexy, sleep paralysis and hypnagogic hallucinations). [37,105,106,107,108,109,110,111,112,113]. Poor sleep quality is associated with impaired immunocompetence and contributes to disease progression [114,115]. Studies have demonstrated a prevalence of sleep-related complaints in LB patients at 100% [37], 96% (LB-C) [12], 92% [88], 82% [86] and 66% (With verification a LB diagnosis was made by a health care provider, that also included copies of positive blood work reports and/or documentation in the patient’s medical record supporting the diagnosis.) [116]. Among (LB-H) patients 82% had vivid nightmares [12].

### 3.11. Addiction

Although there is very little data available on the association between addiction and LB, it is an area that deserves attention since chronic pain and psychiatric illness are seen with LB. Chronic pain from LB (headaches, neurological, musculoskeletal) occurred in 24% of United States confirmed and treated patients and the severity is comparable to post-surgical pain [117,118] Based upon a patient registry of 3900 patients 26% of Lyme patients are administered prescription pain medications compared to 16% of the age matched controls in the general US population [119,120]. The prevalence of chronic pain in suicidal LB patients is 65%, in suicidal and homicidal LB patients the prevalence is 57% and in LB patients who are not suicidal or homicidal the prevalence is 35% [48]. Some LB patients with chronic pain are treated with prescription pain medications, which may include opioids [121,122]. In addition, 51.4% (60 million prescriptions) are received by adults who have a mental health disorder [123]. Unrecognized and inadequately treated mental and physical illnesses are well recognized risks of substance abuse. The prevalence of substance abuse in LB patients is 33% (LB-H) [12], 28% (suicidal and homicidal) [48] and 10% (LB-C) [12]. It is understandable that some LB patients who have been inadequately diagnosed and treated may develop impaired dopamine functioning, become anhedonic, have significant disease progression and self-medicate their psychiatric symptoms and pain, then become dependent, lose a sense of purpose and engage in drug-seeking behavior with benzodiazepines, hypnotics, alcohol, pain medication and marijuana and a few cases which have addressed substance use associated with LB have been reported [124,125,126,127,128,129,130,131].

Also, some LB patients are alcohol sensitive and self-report having a strong effect from a relatively small amount of alcohol (44%) (LB-H) and (24%) (LB-C) [12]. Some LD patients are drug sensitive and can demonstrate toxic symptoms with exposure to minimal doses of prescription psychotropic medications as well as drugs with abuse potential [132]. This drug sensitivity should not be confused with Jarisch-Herxheimer reactions in which there may be an exacerbation of somatic and/or neuropsychiatric symptoms in response to antibiotic treatment [9,24]

### 3.12. Cognitive Impairments

Studies with different study designs reported a number of acquired cognitive impairments in LB/TBD patients [10,132,133,134,135,136,137,138,139,140,141]. The prevalence of these impairments in LB patients are encephalopathy (89%) and memory loss (81%) [91]; attention/concentration impairments (77%), memory complaints (65%) and mental fatigue (70%) (children) [92]; attention and concentration impairments (77%), memory complaints (65%), mental fatigue (70%), cognitive impairment (92%) [116]; memory loss (63%), poor concentration (60%), difficulty finding words (46%), confusion (44%) and inattention (44%) [88]; a conference presentation with impairments of reasoning (93%), memory (92%) and attention (91%) with speaking (75%), listening (73%), reading and/or writing (79%) [142]; short-term memory problems (94%), schoolwork deterioration (94%), brain fog (88%), distractibility (82%), word-finding problems (82%), and moderate to severe sensory hypersensitivity to sound (58%) and/or light (74%); word-finding problems (79%) (children) [86]; memory impairments (76%), processing impairments (78%), dyslexia symptoms (68%) (LB-C) [12] and impaired capacity for sustained and/or selective attention (98%), auditory hyperacusis (88%), sensory hypersensitivity to light, touch, and/or smell (86%), memory impairments which were most commonly working memory and short-term memory (98%), processing impairments (94%), dyslexia symptoms (78%), and executive functioning impairments (98%) (LB-H) [12].

### 3.13. Dementia

There are over 60 articles that address the causal association between LB and dementia [2]. Two of the three basic types of *B. burgdorferi* infections can contribute to a more rapidly developing dementia—the meningovascular form with cerebrovascular infarcts and the atrophic form with meningoencephalitis, cortical atrophy and gliosis. The atrophic form is associated with a more rapidly progressive dementia [22,23,143]. Infection outside the CNS causing immune effects within the CNS can be associated with a very slowly progressive dementia [2,24]. A study of Alzheimer’s brains looking for polymicrobial infections using a polyclonal antibody against *Borrelia* detected structures that appeared not related to spirochetes, but rather to fungi. These structures were not found with a monoclonal antibody. Also, *Borrelia* DNA was undetectable by nested polymerase chain reaction in the ten patients analyzed [144]. Another study of the brains of ten clinically and neuropathologically confirmed Alzheimer’s cases demonstrated spirochetes in the blood, brain, and cerebrospinal fluid of all patients and cultivation revealed three of these patients had *B. burgdorferi* sensu stricto and serological analysis confirmed that these Alzheimer’s patients had Lyme neuroborreliosis [145]. One article states pure Lyme dementia exists, is rare, has a good outcome after antibiotics treatment and is diagnosed with a positive intrathecal anti-*Borrelia* index, however these impressions were not confirmed by brain autopsies [146]. Another study found no geographical correlation between LD and death due to Alzheimer’s disease [147]. A similar debate occurred over 100 years ago regarding the cause of general paresis which was proven when Noguchi and Moore demonstrated *Treponema pallidum* in brain autopsies of general paresis patients [148]. The many similarities between syphilis and LB are noted, brain autopsy studies with direct detection methods are the best approach to research this issue is with autopsy studies with direct detection methods which have demonstrated an association between LB and dementia [22,149,150,151]. In one study when using direct detection methods *B. burgdorferi* was detected in the brain in 25.3% of Alzheimer’s disease cases and was found 13 times more frequently in Alzheimer’s disease cases compared to controls [152].

### 3.14. Seizure Disorders

A number of articles have documented an association between LB and seizures [79,153,154,155,156,157,158,159]. Seizures have also been documented associated with *Bartonella* [155]. Seizure disorders are more common when there is a lengthy delay in diagnosis and effective treatment. Most commonly the seizures are complex partial seizures with significant postictal confusion and are sometimes referred to psychiatrists because they are misdiagnosed as being “psychogenic” or so called “pseudoseizures.” The prevalence of seizures in (LB-H) patients is 20% and they were mostly complex partial seizures [12].

### 3.15. Suicide and Violence

Suicidality seen in LB contributes to causing a significant number of previously unexplained suicides and is associated with immune-mediated and metabolic changes resulting in psychiatric and other symptoms which are probably worsened by negative attitudes about LB from others. Some LB suicides are associated with being overwhelmed by multiple debilitating symptoms, and may be impulsive, bizarre, and unpredictable. Negative attitudes about LB from family, friends, doctors, and the health care system appeared to also contribute to suicide risk. By indirect calculations, it is estimated there are possibly over 1,200 LB suicides in the US per year [48]. In late stage LB the prevalence of suicidality is 43% [48] 46% in (LD-C) and 98% in (LB-H) patients [12]. Posttreatment LB patients meeting CDC surveillance criteria, previously with at least 3 weeks of intravenous antibiotic therapy, with a positive IgG Western blot and persistent post treatment symptoms reported suicidal ideation with a prevalence of 19.8% [160]. Among the patients with moderate-to-severe depression, suicidal ideation was more common with a prevalence of 63.2% [160].

Although most LB patients have no aggressiveness tendencies or mild impairments of frustration tolerance and irritability and pose no danger, a lesser number of patients experience explosive anger, a lesser number of patients experience homicidal thoughts and impulses and much lesser number commit homicides. In a study of 100 LB patients with neuropsychiatric symptoms, none had legal difficulties pre-infections, while 4% (LB-C) and 42% (LB-H) had legal difficulties post-infection [12]. When homicides have occurred, they have been associated with predatory aggression, poor impulse control and psychosis but mostly predatory aggression. The LB patients who committed homicides were evaluated after being contacted by attorneys requesting expert witness opinion. None had long-standing histories of anti-social behavior pre-infection, but instead experienced ego-dystonic intrusive thoughts and impulses post infection [12].

Since such large numbers are affected by LB, a very small percent of patients with these impairments can be highly significant. Most aggression with LB was impulsive, sometimes provoked by intrusive symptoms, sensory stimulation or frustration and the aggressive behavior was invariably bizarre and senseless. LB and the associated immune, biochemical, neurotransmitter, and neural circuit reactions to them can cause impairments that increase the risk of violence. In late stage LB the prevalence of homicidality is 9.6% [12].

### 3.16. Other Psychiatric Findings

Other psychiatric findings caused by LB include mood swings (47%), irritability (47%) [88], anhedonia (56%) (LB-C), anhedonia (86%) (LB-H), exaggerated startle reflex (66%) (LB-C), exaggerated startle reflex (84%) (LB-H), disinhibition (32%) (LB-C), disinhibition (84%) (LB-H), nightmares (58%) (LB-C), nightmares (82%) (LB-H), depersonalization (52%) (LB-C), depersonalization (71%) (LB-H), dissociative episodes (12%) (LB-C) dissociative episodes (38%) (LB-H), derealization (24%) (LB-C), derealization (37%) (LB-H), decreased libido (44%) (LB-C), decreased libido (80%) (LB-H), abrupt mood swings (66%) (LB-C), abrupt mood swings (94%) (LB-H), a decline in social functioning (91%) (LB-H), a decline in school work or work productivity (90%) (LB-H), marital and/or family problems (80%) (LB-H) and legal problems (42%) (LB/-H) [12].

### 3.17. Fatalities

While many LB fatalities, both recognized and unrecognized, may be associated with suicides, drug overdoses, homicides and accidents from cognitive impairments, there are other LB fatalities. Fatalities associated with other neuropsychiatric conditions include congenital Lyme infections, Lyme meningitis, symptomatic late Lyme neuroborreliosis, late Lyme neuritis or neuropathy, meningovascular and neuroborreliosis with cerebral infarcts, intracranial aneurysm, late Lyme encephalitis, late Lyme meningo-encephalitis or meningomyelo encephalitis, atrophic form of Lyme meningo- encephalitis with dementia and subacute presenile dementia. Fatalities associated with somatic impairments include Lyme nephritis, Lyme hepatitis, Lyme aortic aneurysm, coronary artery aneurysm, late Lyme endocarditis, Lyme carditis, late Lyme disease of liver and other viscera, late Lyme disease of kidney & ureter and late Lyme disease of bronchus & lung [49]. 

### 3.18. Assessment

Screening assessments are advisable when evaluating psychiatric symptoms when the possibility of LB may be present [8]. Screening questions include:

- Do you live, vacation or engage in activities in areas that may expose you to ticks?
- Have family members, neighbors, or the family dog been infected?
- Is there a history of a tick bite, possibly with a flu-like illness and/or a bull’s eye or other rash?
- Is there a point at which your health declined, followed by a fluctuating progression and development of multi-systemic symptoms, including cognitive, psychiatric, neurological, and somatic symptoms adversely impacting school, social life, family life?
- Have you ever been treated for Lyme disease, suspected you had Lyme disease but was told it was ruled out?
- Have antibiotics ever caused a sudden worsening followed by an improvement of symptoms?

If the screening assessment increases diagnostic suspicion a further assessment is indicated. LB is diagnosed just like any other neuropsychiatric condition by a comprehensive psychiatric clinical exam relevant to patient’s complaints and findings with a thorough history, mental status exam, review of systems, neurological exam, physical exam, a knowledgeable interpretation of laboratory findings, pattern recognition and clinical judgment. In considering the diagnosis it is important to look for relapsing progressive multi-systemic symptoms, including cognitive, psychiatric, neurological, and somatic symptoms and to remember the greater the multisystemic comorbidity, the greater the likelihood of a condition impacting the entire body such as a complex infectious disease. The presence of a comorbid condition does not rule out the presence of LB [48,161,162,163].

A comprehensive assessment includes an assessment of the following:

- Cognitive: Attention (sustained attention, allocation of attention, distracted by frustration), hypersensitivity (auditory, visual, tactile, olfactory); inability to filter sensory input resulting in stimulation overload; memory (working memory, working spatial memory, short-term memory, long-term memory, word retrieval, number retrieval, name recall, facial recognition, procedural memory, geographical memory); processing (slow processing, letter reversals, spelling errors, word substitution errors, number reversals, reading comprehension impairments, auditory comprehension impairments, sound localization impairments, spatial perceptual distortions, optic ataxia, impaired transposition of laterality, left-right confusion, impaired calculation abilities, impaired fluency of speech, stuttering, slurred speech, impaired fluency of writing, impaired handwriting); executive functioning (unfocused concentration, brain fog, prioritizing multiple tasks, multitasking, racing thoughts, intrusive thoughts, obsessive thoughts, mental apathy, abstract reasoning impairments, time management impairments)
- Imagery: depersonalization, derealization, capacity for visual imagery, hypnagogic hallucinations, vivid nightmares, illusions (auditory, visual), hallucinations (auditory, especially musical, visual, olfactory, sensory).
- Emotional: decreased frustration tolerance, abrupt mood swings, hypervigilance, paranoia, anhedonia
- Behavioral: disinhibition, exaggerated startle reflex, explosive anger, suicidal, homicidal, accident prone, decreased social functioning, decreases school or job productivity, family and marital conflicts, substance abuse, legal difficulties, dissociative episodes, compensatory compulsions, dropping objects, crying spells, self-mutilation
- Psychiatric syndromes: depression, rapid cycling bipolar illness, panic disorder, obsessive compulsive disorder, social anxiety disorder, generalized anxiety disorder, posttraumatic stress disorder
- Sleep disorders: non-restorative sleep, early insomnia, middle of night insomnia, early morning insomnia, excessive daytime sleepiness, loss or reversal of circadian rhythm, restless leg, paroxysmal nocturnal limb movements, sleep apnea (central and/or obstructive), sleep paralysis, hypnagogic hallucinations, sleep attacks, cataplexy, narcolepsy
- Eating disorders: anorexia, weight loss, emotional overeating, carbohydrate craving, weight gain (with or without increased food intake)
- Sexual: decreased libido, increased libido, decrease capacity for arousal, decreased capacity for orgasm, altered sexual imagery
- Temperature control: body temperature fluctuations, flushing, intolerance to heat, intolerance to cold, decreased body temperature, low grade fevers, night sweats, chills
- Headaches: cervical radiculopathy, migraine, thunderclap, tension, cluster, sinus, scalp tenderness, temporal mandibular joint, coital cephalalgia
- Cranial nerves: I: loss of smell, altered taste; II/eye: blurred vision, photophobia, intolerance of fluorescent or flickering light, floaters, flashes, conjunctivitis, eye pain, dry eyes, blind spots, night blindness, peripheral shadows, panopsia, papilledema, iritis, uveitis, optic neuritis; II, IV, VI: double vision, eye drifts when tired, ptosis; V: sensory loss and/or pain in any of the three branches on either side; VII: Bell’s Palsy; VIII: tinnitus, hearing loss, dizziness, vertigo, motion sickness, Tulio’s sign, mal de debarquement; IX, X: episodic loss of speech, choking on food, difficulty swallowing; XI: sternocleidomastoid, trapezius pain and/or weakness; XII: tongue deviates to side
- Seizures: complex partial, grand mal
- Neuropathy: numbness, tingling, sensory loss, burning, crawling sensation (formication), static electricity sensation, stabbing sensation, weakness
- Other neurological: fatigue, tremor, twitching, muscle tightness, myoclonic jerks, tics, Tourette’s, ataxia, spasticity, meningismus, disc disease, positive Romberg, postural tachycardia syndrome (POTS), ortho static hypotension, gait disturbances, spinal cord signs, white matter lesions, sensation of vibration
- Musculoskeletal: Joint pain, migratory joint pain, swelling, tightness, crepitations, neck and back discomfort; periostitis and bone tenderness of tibia, ribs, iliac crest, sternum, clavicle; epicondylitis; plantar fasciitis, foot tenderness; fibromyalgia; myalgia, costochondritis (ear, nose, costochondral junctions, xyphoid); tendonitis; carpal tunnel syndrome
- Cardiac: chest pain, heart block, irregular heart rate, mitral valve prolapse, racing pulse, POTS, pericarditis, cardiomyopathy, murmur, hypertension, hypertensive crisis
- Pulmonary/upper respiratory: shortness of breath, air hunger, cough, sore throats, swollen glands, asthma
- Gastrointestinal: Reflux, irritable gut, nervous stomach, irritable bowel, abdominal bloating, reduced gastrointestinal motility, gastroparesis, cholecystitis, gall stones
- Genitourinary: Spastic bladder, genital pain (testicular/pelvic), menstrual irregularity, breast tenderness, sexual dysfunction, decreased libido, urinary incontinence, interstitial cystitis
- Immune: fevers, sweats, chills
- Other: alcohol intolerance, hair loss, thyroid disease, adrenal insufficiency, hypoglycemia, ankle edema, tooth pain, periodontal disease, nose bleeds, ecchymoses, splenomegaly, multiple chemical sensitivities, allergies, lymphocytoma, stria, acrodermatitis chronicum atrophicans

The more common symptoms seen in LB include poor attention span, being easily distracted by frustration, sensory hypersensitivity causing patients to feel overwhelmed, poor short-term memory, dyslexia symptoms, slow processing, executive dysfunction, brain fog, poor time management, depersonalization, intrusive images and thoughts, musical hallucinations, low frustration tolerance, abrupt mood swings, impulsivity, paranoia, explosive anger, suicidality, anhedonia, decreased productivity, depression, long duration panic attacks, social anxiety, generalized anxiety, obsessiveness, non-restorative sleep, appetite disturbances, decreased libido, headaches, cranial nerve symptoms, neuropathy, autonomic nervous system symptoms, musculoskeletal symptoms, gastrointestinal symptoms, genitourinary symptoms, cardiovascular symptoms, fatigue, chronic pain and alcohol intolerance [12,48,161].

After an adequate clinical assessment is performed, laboratory testing with proper interpretation may add to the assessment. The results of laboratory testing are dependent upon the stage of the illness [10] and the unreliability of laboratory testing for *B. burgdorferi* contributing to the underdiagnosis of LB and progression to psychiatric illness has been recognized [11]. Although direct testing methods (culture, DNA/polymerase chain and antigen testing) correlates with active infection, indirect immune based testing is more readily available and the two-tier CDC surveillance criteria is most commonly used. The CDC has stated “This surveillance case definition was developed for national reporting of Lyme disease; it is not intended to be used in clinical diagnosis” and no evidence has ever been submitted to explain any subsequent change in this position [164]. In addition, meta-analyses and other research demonstrate significant limitations of the sensitivity of testing [165,166,167,168,169,170]. Immune reactivity alone does not differentiate whether there is a current or a past infection. However, positive testing in the past or present and immune reactivity to specific bands on the Western blot indicates exposure at some point to *B. burgdorferi.* Immune reactivity combined with multisystemic disease progression is more supportive of current active infection. The American Psychiatric Association guidelines recognize the need to consider LB in the etiology of psychiatric illness, but do not address laboratory testing; the current International Lyme and Associated Diseases Society guidelines address treatment, but not laboratory testing and the Infectious Diseases Society of America guidelines and other guidelines based upon them do not address psychiatric manifestations of LB [171,172,173]. Prior review articles addressing the neuropsychiatric manifestations of LB have addressed testing in LB patients with neuropsychiatric symptoms [9,10,11]. Improved tests are being developed [174]. As controversies surrounding testing continue, it is important for physicians to keep current with improvements in testing and to remember that no test currently has the capacity to rule out the possibility of LB [167,170]. The differential diagnosis is complex but the more common differential diagnosis includes other chronic systemic conditions and infections, since many of the symptoms and syndromes seen with LB may overlap with conditions other than LB [175].

If an inadequate clinical exam is performed it can result in viewing the symptoms as being vague and subjective. Caution must be used in considering the symptoms as having a psychogenic basis, such as hypochondriasis, somatization disorder, or a psychosomatic condition. Both hypochondriasis and psychosomatic illnesses begin in childhood and are lifelong conditions with a psychodynamic explanation and vary in intensity depending upon life stressors. If a complex, progressive multisystemic illness begins in a person who had been reasonably healthy throughout most of their life, the likelihood that this is psychosomatic or has some other psychogenic basis is very remote. Another diagnostic error by clinicians who lack psychiatric diagnostic capability is to consider these symptoms as being so called “medically unexplained symptoms” or “bodily distress syndrome.” The concept of medically unexplained symptoms was removed from the most recent DSM (DMS-5) since these symptoms were often instead medically unexamined symptoms and the concept of bodily distress syndrome is not included in the DSM-5 and lacks objective criteria [3].

### 3.19. Treatment

All treatments are a risk vs. benefit decision and inadequately treated LB can result in a broad spectrum of risks as previously described. A complex, chronic, LB patient may have a multitude of different symptoms. What causes a condition may be different from what perpetuates a condition. It is best to make a list with the patient ranking which symptoms are the most severe and most impede recovery and consider how the symptoms interact with each other. This will determine the sequence of initiating different treatment strategies. One major question is considering whether antibiotic or symptomatic treatment has higher priority. When a patient has been treated with just antibiotics and has not adequately responded, consider treating the symptoms with psychotropics or other symptomatic treatments. When a patient has been treated with just psychotropics and has not adequately responded, consider treating the symptoms with antibiotics [176,177]. When a patient is treatment resistant consider both symptomatic and antibiotic treatment [176,177].

Although each patient may have a unique presentation, the most common symptoms impeding recovery are non-restorative sleep and/or chronic unremitting stress. Both are associated with a high allostatic load and compromised immune functioning. Non-restorative sleep is often associated with the terrible triad which consists of non-restorative sleep, fatigue and cognitive impairments [114,115,176].

Chronic unremitting stress is often associated with hyperarousal and emotional symptoms such as depression, anxiety, depersonalization, mood swings and psychosis. Other symptoms that may be a focus of treatment may include chronic pain (headaches, neuropathy, radiculopathy, musculoskeletal, etc.), complex partial seizures, dysautonomia, gastrointestinal symptoms, genitourinary symptoms, substance abuse and addiction [176].

Regardless of the debate surrounding the chronicity of infection and the chronicity of symptoms with LB, treating psychiatric symptoms with psychotropics can prevent and sometimes reverse disease progression. Since non-restorative sleep and chronic unremitting stress contribute to compromised immune functioning and disease progression, remediating these symptoms can improve immune functioning and resistance to infection which may reduce disease progression and contribute to recovery. Successful psychiatric management can sometimes result in reduction of infection and successful reduction of infection can sometimes result in reducing psychiatric symptoms and reducing the need for psychotropics [176].

No drugs are specifically approved by the Federal Drug Administration (FDA) for the treatment of psychiatric symptoms associated with LB. Since LB can be associated with the full spectrum of psychiatric symptoms, all psychotropic are sometimes used and these medications may or may not be FDA approved to treat the relevant symptom [176].

Separate and apart from the potential benefit of psychotropics upon mental symptoms when used as psychotropics, some also have antimicrobial, immune and other effects [178].

When the symptoms are caused by persistent relapsing infection, antibiotic treatment late in the course of the illness may prevent some further neuropsychiatric disease progression but may be unable to reverse all the previously established neuropsychiatric impairments. Since our current technological limitations prevent us from being sure *B. burgdorferi* and all other tick-borne infections have been eradicated by antibiotic treatment, after stabilization constant vigilance is needed to recognize a possible relapse that may require further treatment [176].

## 4. Discussion

It is recognized there is a selection bias in many of the studies discussed in this article since only the more severe cases were referred to the mental health professionals who specialized in treating LB. Also, a significant amount of this article and many of the citations are based upon clinical observations, publications and presentations by the author. Critical appraisal and research by others to independently validate, modify or refute the author’s findings is needed to advance future research and a better understanding of the content of this article.

In reviewing the different studies, the greatest level of current debate exists regarding whether or not there is a causal association between LB and developmental disabilities, ASD, schizophrenia and dementia. Very little data exists on the association between LB and social anxiety and substance abuse.

Although there is evidence supporting a causal association between psychiatric findings and LB, controversy does exist, and it is necessary to also consider articles negating this association.

One study concluded their mean summary assessment scores of the mental health of LB patients 11–20 years post infection was similar to those of the general population [179]. However, there were multiple flaws in the study that included a failure to perform psychiatric assessments, inclusion of only 35% of the initial study group which suggests a selection bias, the use of assessment scales insufficient to adequately evaluate the cognitive and psychiatric impairments seen in LB, a failure to differentiate between statistical and clinical significance, a research design that was not designed (or powered) a priori to detect differences in functional outcomes, data that did not support the conclusion and the inclusion of only subjects who were effectively diagnosed and treated early [180]. This group of patients are quite different that other studies with greater psychiatric morbidity in which 8 years [48] or 9 years [12] elapsed in the average patient before effective diagnosis and treatment.

Another study concluded from their results that “psychiatric comorbidity and other psychological factors are prominent in the presentation and outcome of some patients who inaccurately ascribe longstanding symptoms to chronic Lyme disease” [181]. In this study the authors identified a group that was labeled as chronic multi-symptom illness or post Lyme disease syndrome, which were patients who met CDC surveillance criteria for Lyme disease and in most cases the two-tiered protocol for laboratory tests and had received what the authors considered adequate prior antibiotic treatment defined by guidelines from the Infectious Diseases Society of America but continued to report persistent symptoms ascribed to Lyme disease [182]. These patients demonstrated significant psychiatric symptoms which included any clinical psychiatric disorder 48.4%, current depression 26.3%, past depression 3.2%, depression/dysthymia 16.1%, anxiety disorder 29%, panic disorder 12.9% and generalized anxiety disorder 25.8% [182]. Their study did not evaluate the mental health of the patients prior to infection. In subsequent studies that looked at the pre-infection psychiatric findings it was clear that most patients had few psychiatric findings pre-infection [12,48]. Therefore, a significant amount of the psychiatric comorbidity described in this article may have been acquired post-infection.

## 5. Conclusions

Recognizing the association between Lyme borreliosis and neuropsychiatric impairments is a major advance in psychiatry.

Lyme borreliosis, possibly with other interactive infections in the body can evade and suppress the immune system and cause immune effects and biochemical changes in the brain causing neuropsychiatric symptoms. Sleep disorders and chronic unremitting stress associated with these impairments contribute to further disease progression of neuropsychiatric symptoms. The pathological effects of these processes may result in developmental disorders, autism spectrum disorders, schizoaffective disorders, bipolar disorder, depression, anxiety disorders (panic disorder, social anxiety disorder, generalized anxiety disorder, posttraumatic stress disorder, intrusive symptoms), eating disorders, sleep disorders, decreased libido, addiction, opioid addiction, cognitive impairments, dementia, seizure disorders, suicide, violence, anhedonia, depersonalization, dissociative episodes, derealization and other impairments.

Diagnosis of Lyme borreliosis is achieved by a screening assessment followed by a thorough history, comprehensive psychiatric clinical exam, review of systems, mental status exam, neurological exam and physical exam relevant to the patient’s complaints and findings with clinical judgment, pattern recognition and knowledgeable interpretation of laboratory findings.

Treatment approaches that reduce symptoms that contribute to disease progression (sleep disorders, fatigue, cognitive impairments, depression anxiety disorders, chronic pain, etc.) in combination with antimicrobial and other treatments can improve recovery.

Future studies to clarify the pathophysiology and look more at the prevalence of these infections in patients with identified neuropsychiatric impairments can improve understanding of the causes of mental illness and violence and result in more effective prevention, diagnosis and treatment.

Sir William Osler, the father of American Medicine said “He who knows syphilis knows medicine.” It can now be said that He who knows Lyme borreliosis knows about medicine, neurology, psychiatry, immunology, psychoimmunology, neurochemistry, ecology, epidemiology, entomology, law, politics, and ethics.
